# Supplementary material for: Evolution of precopulatory and post‐copulatory strategies of inbreeding avoidance and associated polyandry
Source: J Evol Biol. 2017 Nov 3;31(1):31–45. doi: 10.1111/jeb.13189 (PMC5765502; doi:10.1111/jeb.13189)
Supplement: Supplementary file 1 — Figure S1 Individual replicate results given c P = 0, c M = 0, & c F = 0. Figure S2 Individual replicate results given c P = 0, c M = 0:02, & c F = 0. Figure S3 Individual replicate results given c P = 0:02, c M = 0, & c F = 0. Figure S4 Individual replicate results given c P = 0, c M = 0, & c F = 0:02 Figure S5 Individual replicate results given c P = 0:02, c M = 0:02, & c F = 0 Figure S6 Individual replicate results given c P = 0, c M = 0:02, & c F = 0:02 Figure S7 Individual replicate results given c P = 0:02, c M = 0, & c F = 0:02. Figure S8 Individual replicate results given c P = 0:02, c M = 0:02, & c F = 0:02. Figure S9 Relative cost asymmetries 0:01 & 0:03. Figure S10 Relative cost asymmetries 0:01 & 0:02. Figure S11 All cost combinations. Figure S12 Dynamic F a, fixed P a = 1 and M a = −10. [file JEB-31-31-s001.pdf]

Evolution of pre-copulatory and post-copulatory strategies of inbreeding avoidance and associated polyandry

Supplemental results showing change in allele values over different parameter combinations, and allele values over generations for single replicates

Here we present supplemental results from an individual-based model in which alleles underlying polyandry (P_a), pre-copulatory inbreeding strategy (M_a) and post-copulatory inbreeding strategy (F_a) affect evolving phenotypic values for each trait over 40000 generations. Figures S1-S8 show the dynamics of individual replicate simulations for all trait cost combinations of 0 and 0.02; these figures also show correlations of mean allele values among replicates. Figures S9 and S10 show cost asymmetry simulations that do not appear in the main text. Figure S11 summarises trajectories of allele values across replicate simulations for all cost combinations of 0 and 0.02 for polyandry (c_P), pre-copulatory inbreeding strategy (c_M), and post-copulatory inbreeding strategy (c_F). Figure S12 shows how F_a alleles change over generations given that M_a alleles are fixed for strong inbreeding avoidance and P_a alleles are fixed for polyandry.

Table of Contents

Figure S1: Individual replicate results given $c_P = 0$, $c_M = 0$, & $c_F = 0$ **S3**

Figure S2: Individual replicate results given $c_P = 0$, $c_M = 0.02$, & $c_F = 0$ **S4**

Figure S3: Individual replicate results given $c_P = 0.02$, $c_M = 0$, & $c_F = 0$ **S5**

Figure S4: Individual replicate results given $c_P = 0$, $c_M = 0$, & $c_F = 0.02$ **S6**

Figure S5: Individual replicate results given $c_P = 0.02$, $c_M = 0.02$, & $c_F = 0$ **S7**

Figure S6: Individual replicate results given $c_P = 0$, $c_M = 0.02$, & $c_F = 0.02$ **S8**

Figure S7: Individual replicate results given $c_P = 0.02$, $c_M = 0$, & $c_F = 0.02$ **S9**

Figure S8: Individual replicate results given $c_P = 0.02$, $c_M = 0.02$, & $c_F = 0.02$ **S10**

Figure S9: Relative cost asymmetries 0.01 & 0.03 **S11**

Figure S10: Relative cost asymmetries 0.01 & 0.02 **S12**

Figure S11: All cost combinations **S13**

Figure S12: Dynamic F_a, fixed $P_a = 1$ and $M_a = -10$ **S14**

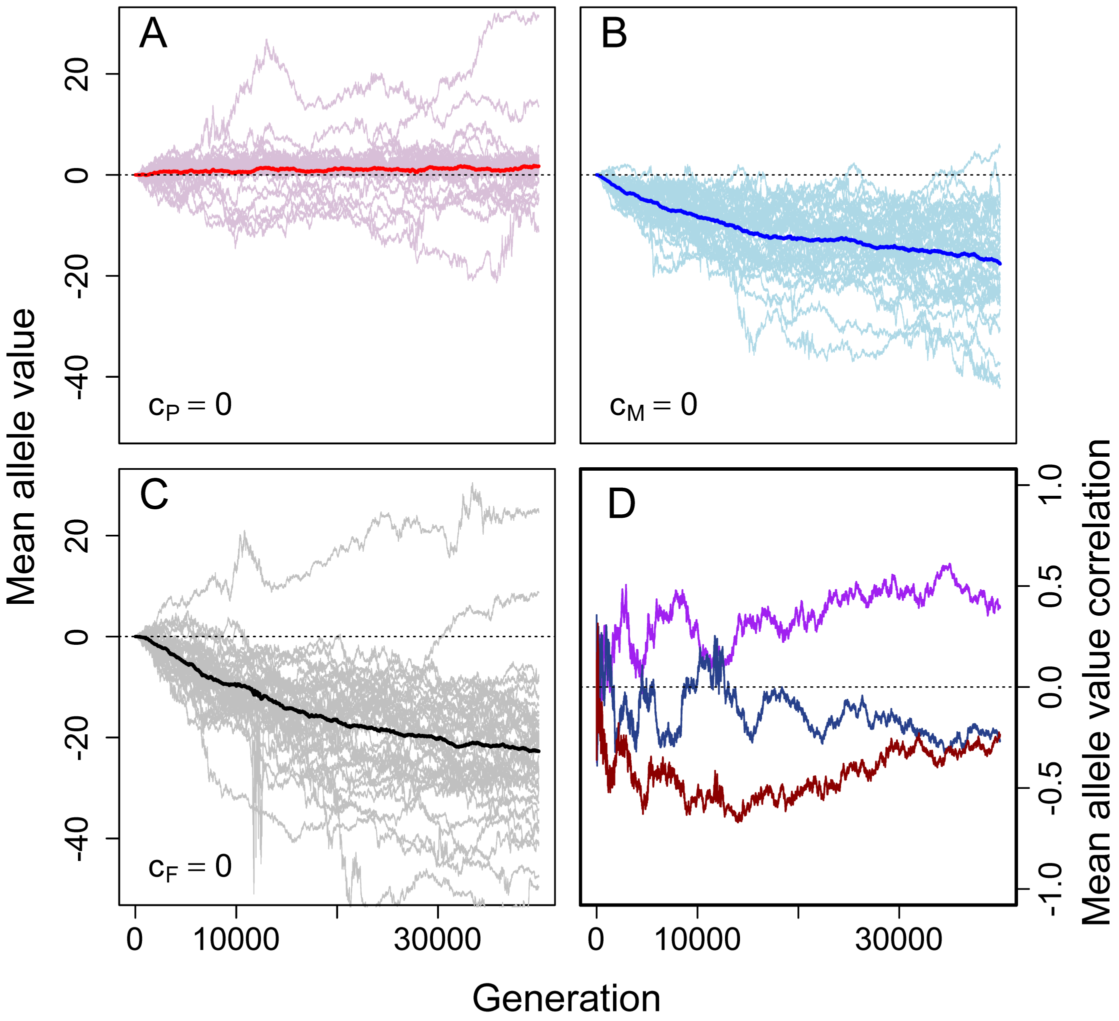

Figure S1: Mean allele values underlying tendency for polyandry (A; red lines), pre-copulatory inbreeding strategy (B; blue lines), and post-copulatory inbreeding strategy (C; black lines), as calculated across all individuals in 40 replicate simulations with identical starting conditions over 40000 generations and given strong inbreeding depression. Thick lines show grand means of replicates, and thin lines show individual replicates. Panels A-C thereby show the same information as in Figure S11A, but with individual replicates replacing standard error around grand mean values. Panel D illustrates among replicate correlations in mean allele values over generations. Purple, dark red, and dark blue lines show correlations between P_a & M_a, P_a & F_a, and M_a & F_a, respectively. Dotted horizontal lines show where the y-axis equals zero.

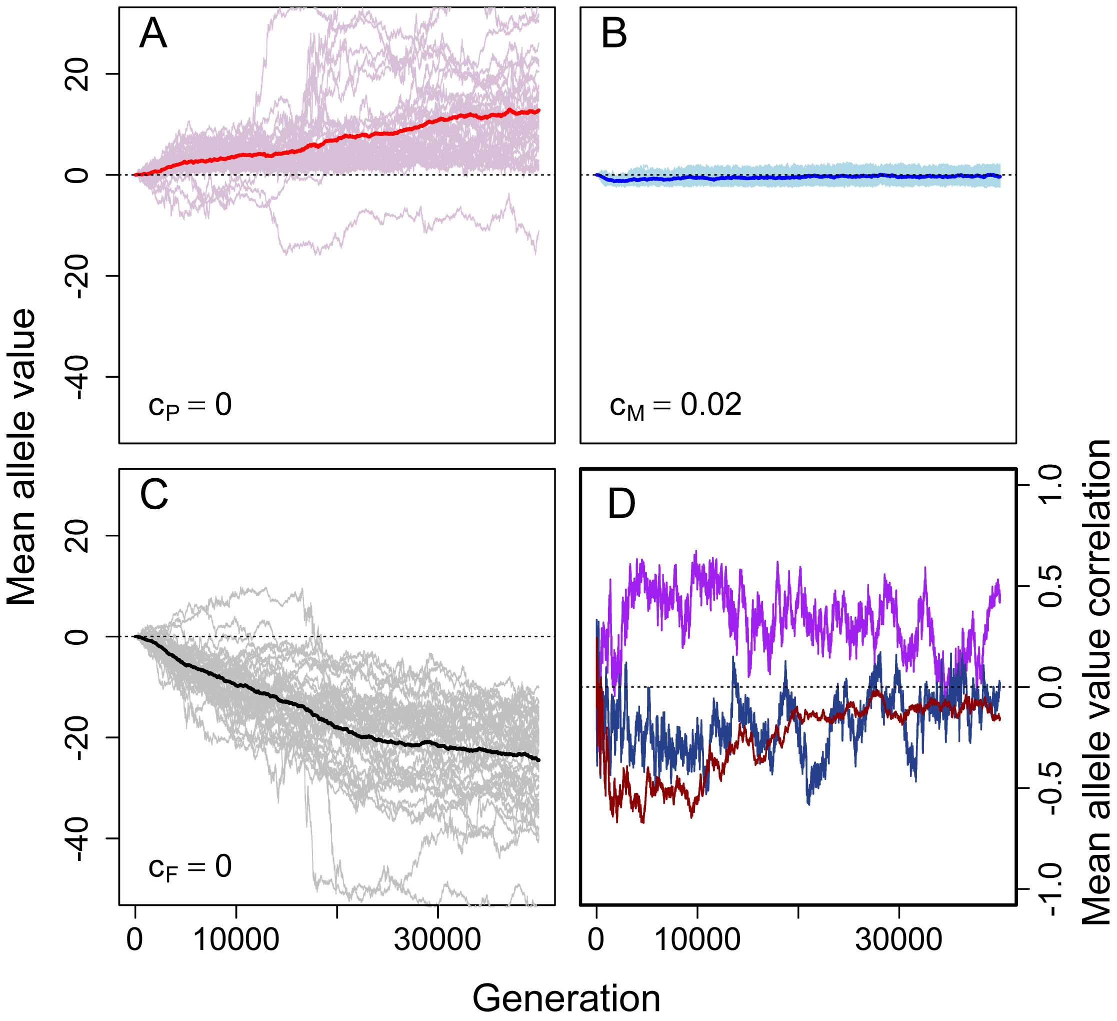

Figure S2: Mean allele values underlying tendency for polyandry (A; red lines), pre-copulatory inbreeding strategy (B; blue lines), and post-copulatory inbreeding strategy (C; black lines), as calculated across all individuals in 40 replicate simulations with identical starting conditions over 40000 generations and given strong inbreeding depression. Thick lines show grand means of replicates, and thin lines show individual replicates. Panels A-C thereby show the same information as in Figure S11B, but with individual replicates replacing standard error around grand mean values. Panel D illustrates among replicate correlations in mean allele values over generations. Purple, dark red, and dark blue lines show correlations between P_a & M_a, P_a & F_a, and M_a & F_a, respectively. Dotted horizontal lines show where the y-axis equals zero.

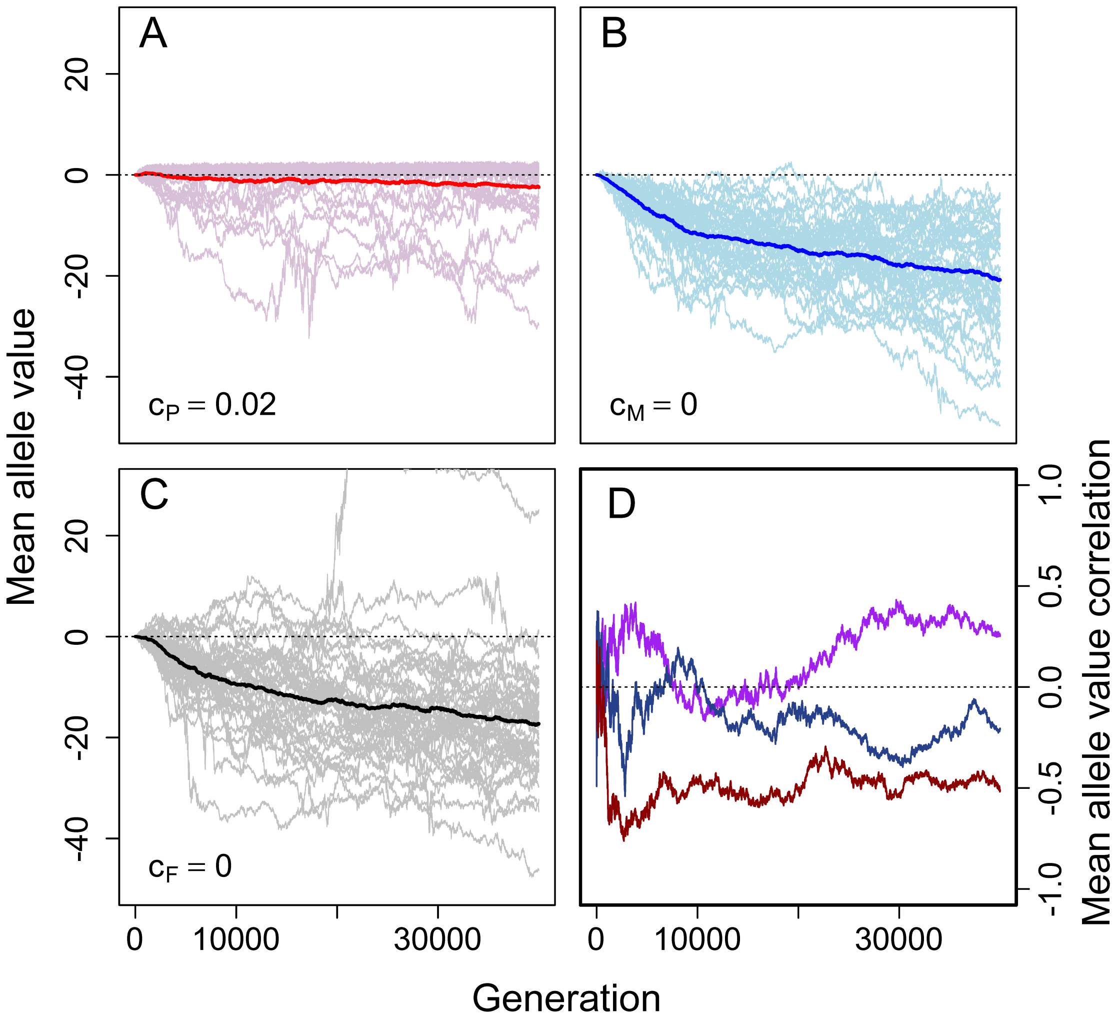

Figure S3: Mean allele values underlying tendency for polyandry (A; red lines), pre-copulatory inbreeding strategy (B; blue lines), and post-copulatory inbreeding strategy (C; black lines), as calculated across all individuals in 40 replicate simulations with identical starting conditions over 40000 generations and given strong inbreeding depression. Thick lines show grand means of replicates, and thin lines show individual replicates. Panels A-C thereby show the same information as in Figure S11C, but with individual replicates replacing standard error around grand mean values. Panel D illustrates among replicate correlations in mean allele values over generations. Purple, dark red, and dark blue lines show correlations between P_a & M_a, P_a & F_a, and M_a & F_a, respectively. Dotted horizontal lines show where the y-axis equals zero.

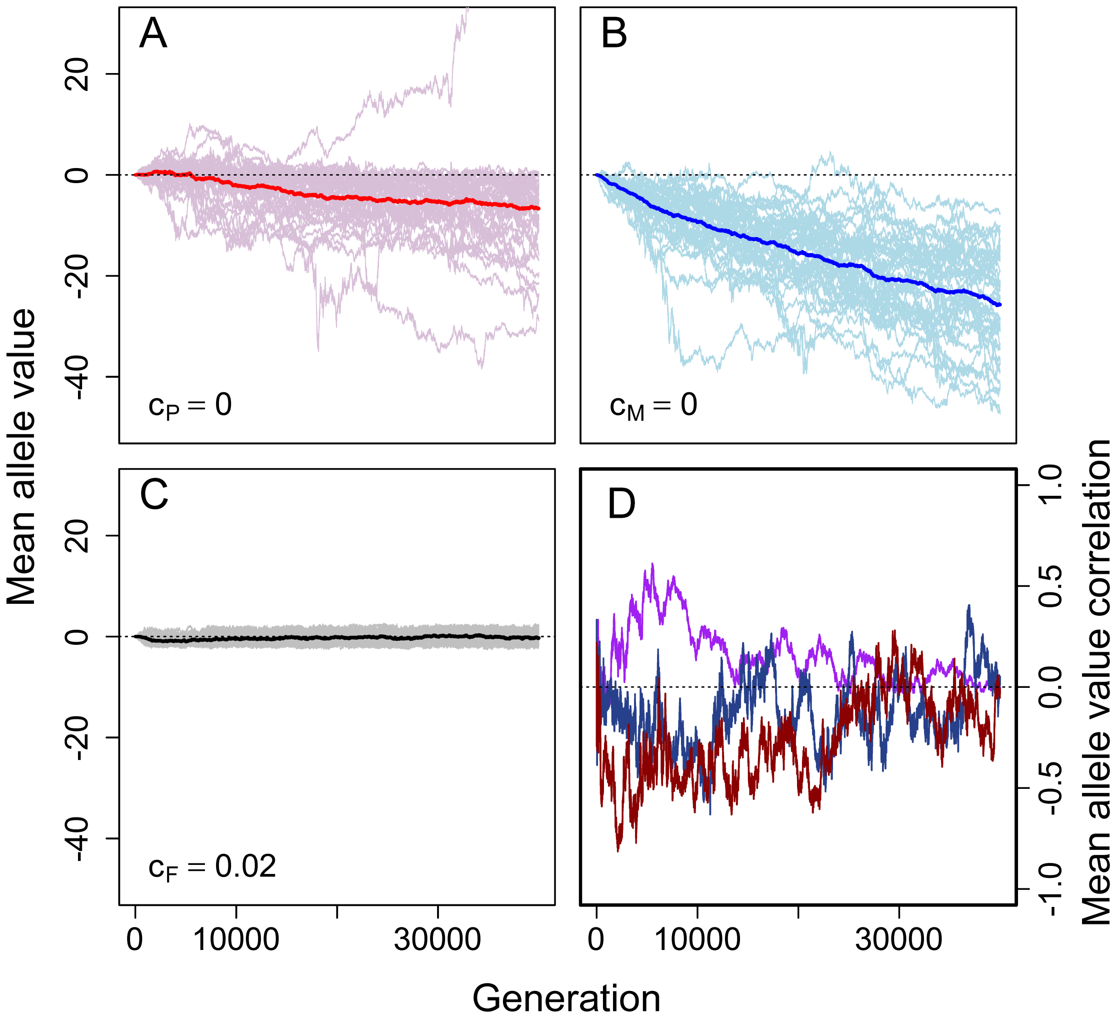

Figure S4: Mean allele values underlying tendency for polyandry (A; red lines), pre-copulatory inbreeding strategy (B; blue lines), and post-copulatory inbreeding strategy (C; black lines), as calculated across all individuals in 40 replicate simulations with identical starting conditions over 40000 generations and given strong inbreeding depression. Thick lines show grand means of replicates, and thin lines show individual replicates. Panels A-C thereby show the same information as in Figure S11D, but with individual replicates replacing standard error around grand mean values. Panel D illustrates among replicate correlations in mean allele values over generations. Purple, dark red, and dark blue lines show correlations between P_a & M_a, P_a & F_a, and M_a & F_a, respectively. Dotted horizontal lines show where the y-axis equals zero.

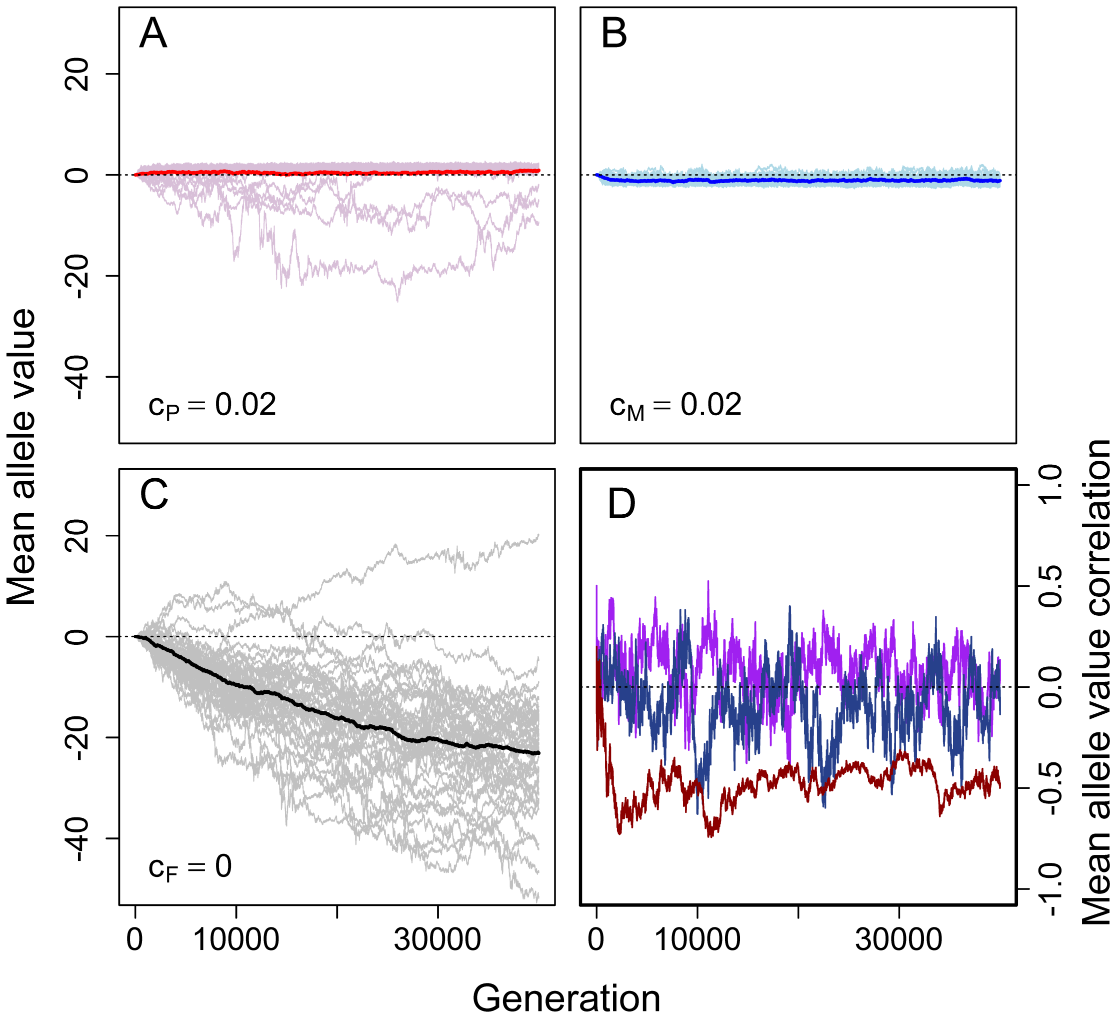

Figure S5: Mean allele values underlying tendency for polyandry (A; red lines), pre-copulatory inbreeding strategy (B; blue lines), and post-copulatory inbreeding strategy (C; black lines), as calculated across all individuals in 40 replicate simulations with identical starting conditions over 40000 generations and given strong inbreeding depression. Thick lines show grand means of replicates, and thin lines show individual replicates. Panels A-C thereby show the same information as in Figure S11E, but with individual replicates replacing standard error around grand mean values. Panel D illustrates among replicate correlations in mean allele values over generations. Purple, dark red, and dark blue lines show correlations between P_a & M_a, P_a & F_a, and M_a & F_a, respectively. Dotted horizontal lines show where the y-axis equals zero.

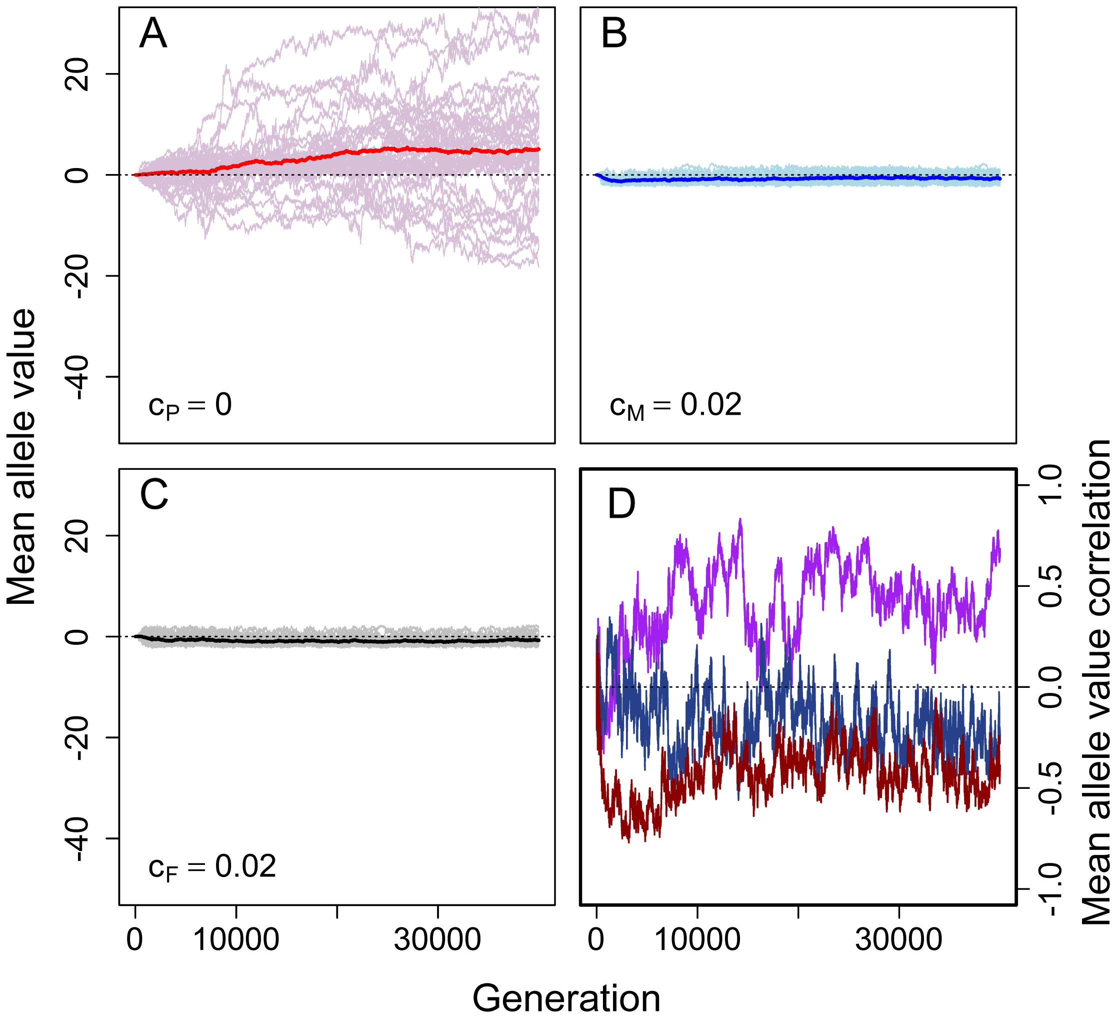

Figure S6: Mean allele values underlying tendency for polyandry (A; red lines), pre-copulatory inbreeding strategy (B; blue lines), and post-copulatory inbreeding strategy (C; black lines), as calculated across all individuals in 40 replicate simulations with identical starting conditions over 40000 generations and given strong inbreeding depression. Thick lines show grand means of replicates, and thin lines show individual replicates. Panels A-C thereby show the same information as in Figure S11F, but with individual replicates replacing standard error around grand mean values. Panel D illustrates among replicate correlations in mean allele values over generations. Purple, dark red, and dark blue lines show correlations between P_a & M_a, P_a & F_a, and M_a & F_a, respectively. Dotted horizontal lines show where the y-axis equals zero.

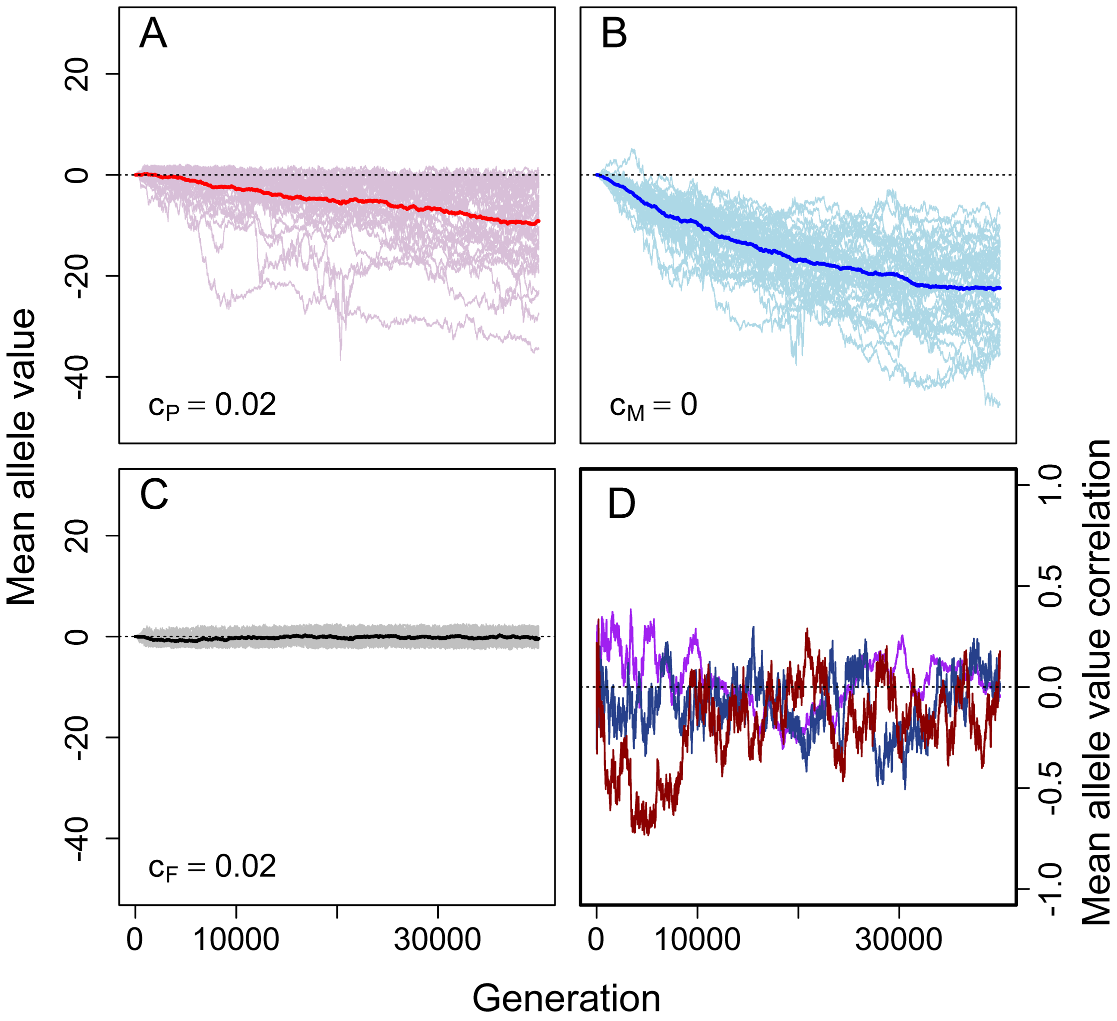

Figure S7: Mean allele values underlying tendency for polyandry (A; red lines), pre-copulatory inbreeding strategy (B; blue lines), and post-copulatory inbreeding strategy (C; black lines), as calculated across all individuals in 40 replicate simulations with identical starting conditions over 40000 generations and given strong inbreeding depression. Thick lines show grand means of replicates, and thin lines show individual replicates. Panels A-C thereby show the same information as in Figure S11G, but with individual replicates replacing standard error around grand mean values. Panel D illustrates among replicate correlations in mean allele values over generations. Purple, dark red, and dark blue lines show correlations between P_a & M_a, P_a & F_a, and M_a & F_a, respectively. Dotted horizontal lines show where the y-axis equals zero.

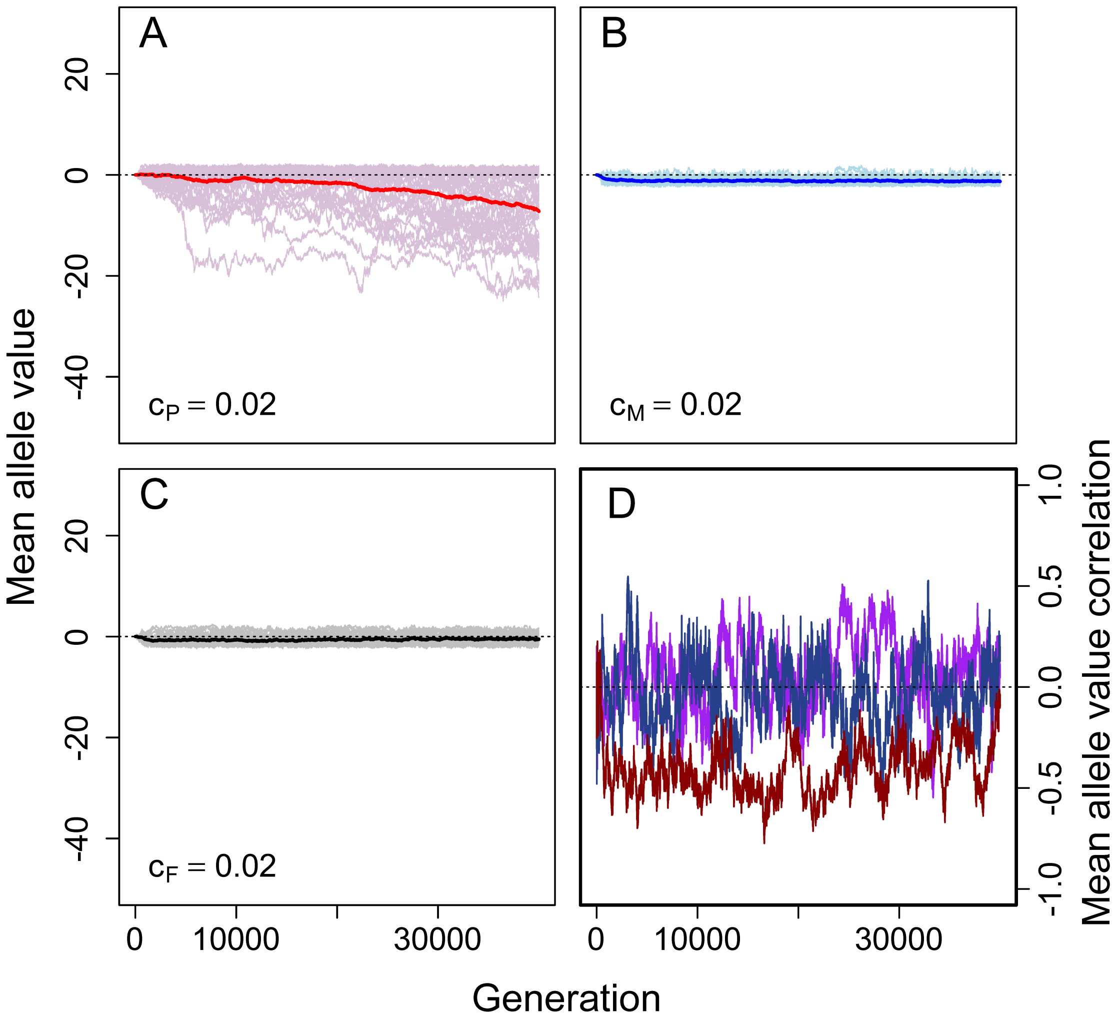

Figure S8: Mean allele values underlying tendency for polyandry (A; red lines), pre-copulatory inbreeding strategy (B; blue lines), and post-copulatory inbreeding strategy (C; black lines), as calculated across all individuals in 40 replicate simulations with identical starting conditions over 40000 generations and given strong inbreeding depression. Thick lines show grand means of replicates, and thin lines show individual replicates. Panels A-C thereby show the same information as in Figure S11H, but with individual replicates replacing standard error around grand mean values. Panel D illustrates among replicate correlations in mean allele values over generations. Purple, dark red, and dark blue lines show correlations between P_a & M_a, P_a & F_a, and M_a & F_a, respectively. Dotted horizontal lines show where the y-axis equals zero.

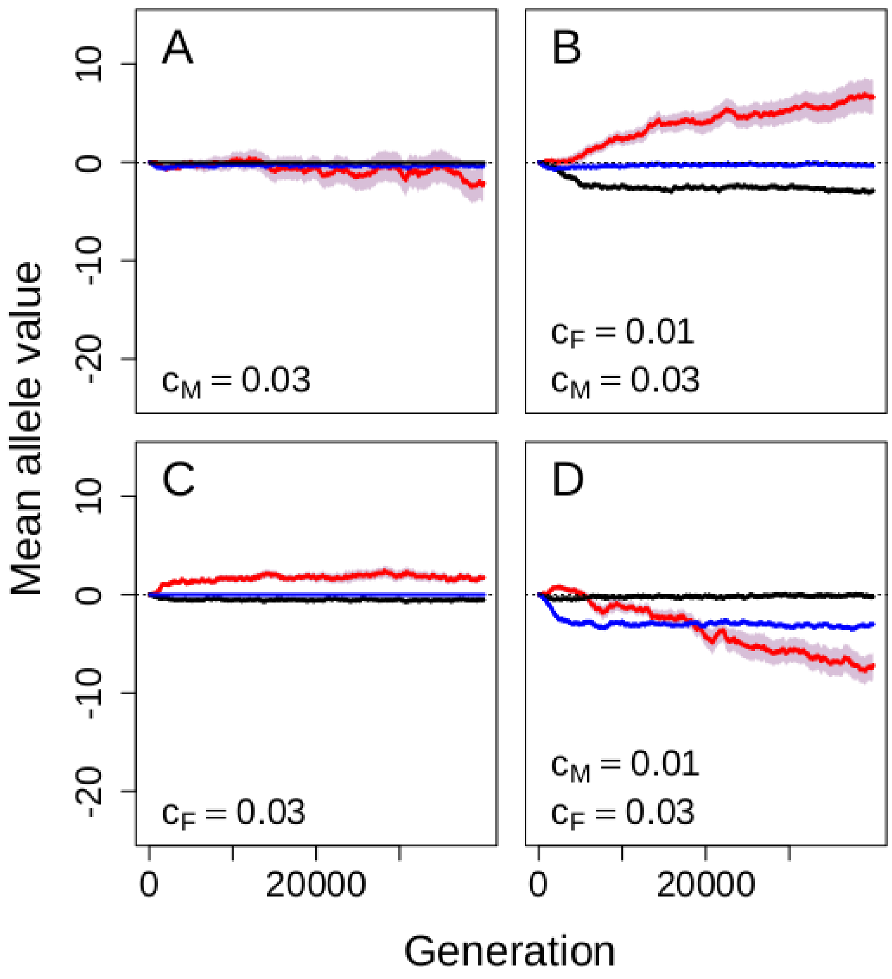

Figure S9: Simulations with asymmetric direct costs, which are different but comparable to Figure 3 of the main text. These supplemental simulations support the general conclusion that costly pre-copulatory and post-copulatory inbreeding strategy allele values become less negative (i.e., closer to zero, indicating reduced inbreeding avoidance) over generations given the co-occurence of a less costly inbreeding strategy. Values in this figure reflect the same amount of cost asymmetry between traits, but with both traits having non-zero direct costs. Mean allele values underlying tendency for polyandry (red), pre-copulatory inbreeding strategy (blue), and post-copulatory inbreeding strategy (black) when (A and B) costly pre-copulatory inbreeding strategy ($c_M = 0.03$) can evolve and post-copulatory inbreeding strategy is (A) fixed for random fertilisation or (B) can also evolve but is less costly ($c_F = 0.01$), and when (C and D) costly post-copulatory inbreeding strategy ($c_F = 0.03$) can evolve and pre-copulatory inbreeding strategy is (C) fixed for random mating or (D) can also evolve but is less costly ($c_M = 0.01$). Mean allele values (solid lines) and associated standard errors (shading) are calculated across all individuals within a population over 40000 generations across 40 replicate populations. Negative mean allele values indicate strategies of inbreeding avoidance or tendency for monandry, and positive values indicate strategies of inbreeding preference or tendency for polyandry. In all panels, polyandry is cost free.

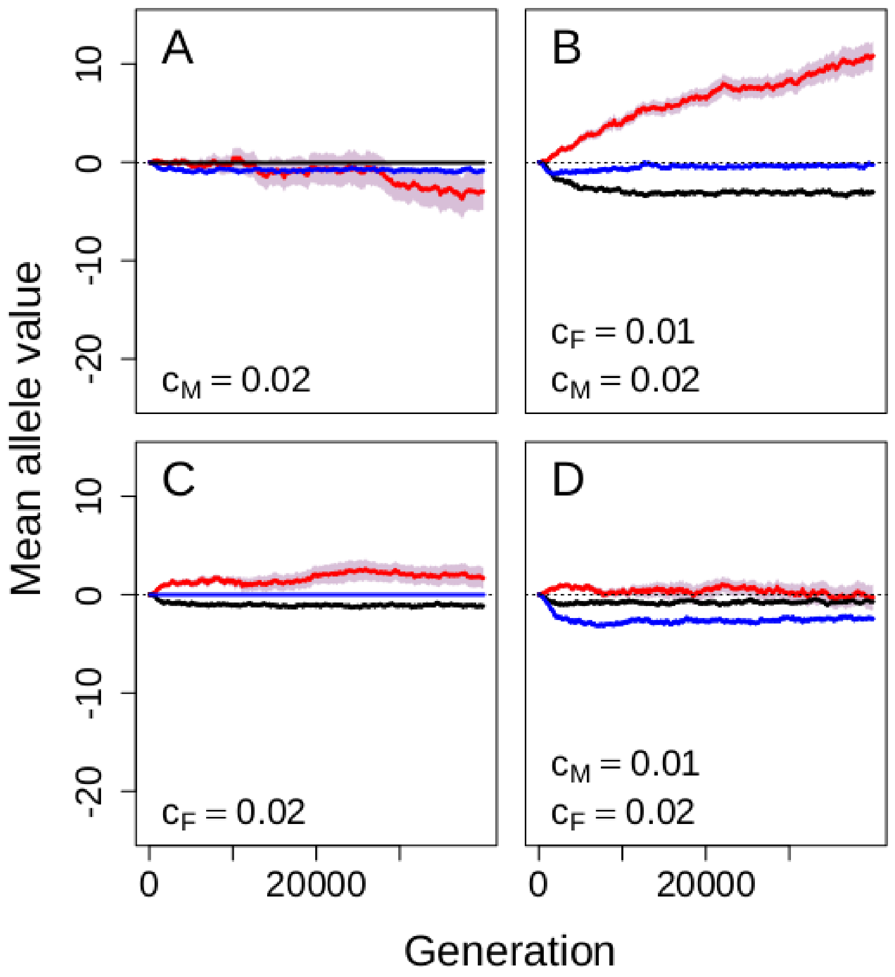

Figure S10: Simulations with asymmetric direct costs, which are different but comparable to Figure 3 of the main text. These supplemental simulations support the general conclusion that costly pre-copulatory and post-copulatory inbreeding strategy allele values become less negative (i.e., closer to zero, indicating reduced inbreeding avoidance) over generations given the co-occurence of a less costly inbreeding strategy. Values in this figure reflect a smaller cost asymmetry between traits than simulated in the main text, with both traits having non-zero direct costs. Mean allele values underlying tendency for polyandry (red), pre-copulatory inbreeding strategy (blue), and post-copulatory inbreeding strategy (black) when (A and B) costly pre-copulatory inbreeding strategy ($c_M = 0.02$) can evolve and post-copulatory inbreeding strategy is (A) fixed for random fertilisation or (B) can also evolve but is less costly ($c_F = 0.01$), and when (C and D) costly post-copulatory inbreeding strategy ($c_F = 0.02$) can evolve and pre-copulatory inbreeding strategy is (C) fixed for random mating or (D) can also evolve but is less costly ($c_M = 0.01$). Mean allele values (solid lines) and associated standard errors (shading) are calculated across all individuals within a population over 40000 generations across 40 replicate populations. Negative mean allele values indicate strategies of inbreeding avoidance or tendency for monandry, and positive values indicate strategies of inbreeding preference or tendency for polyandry. In all panels, polyandry is cost free.

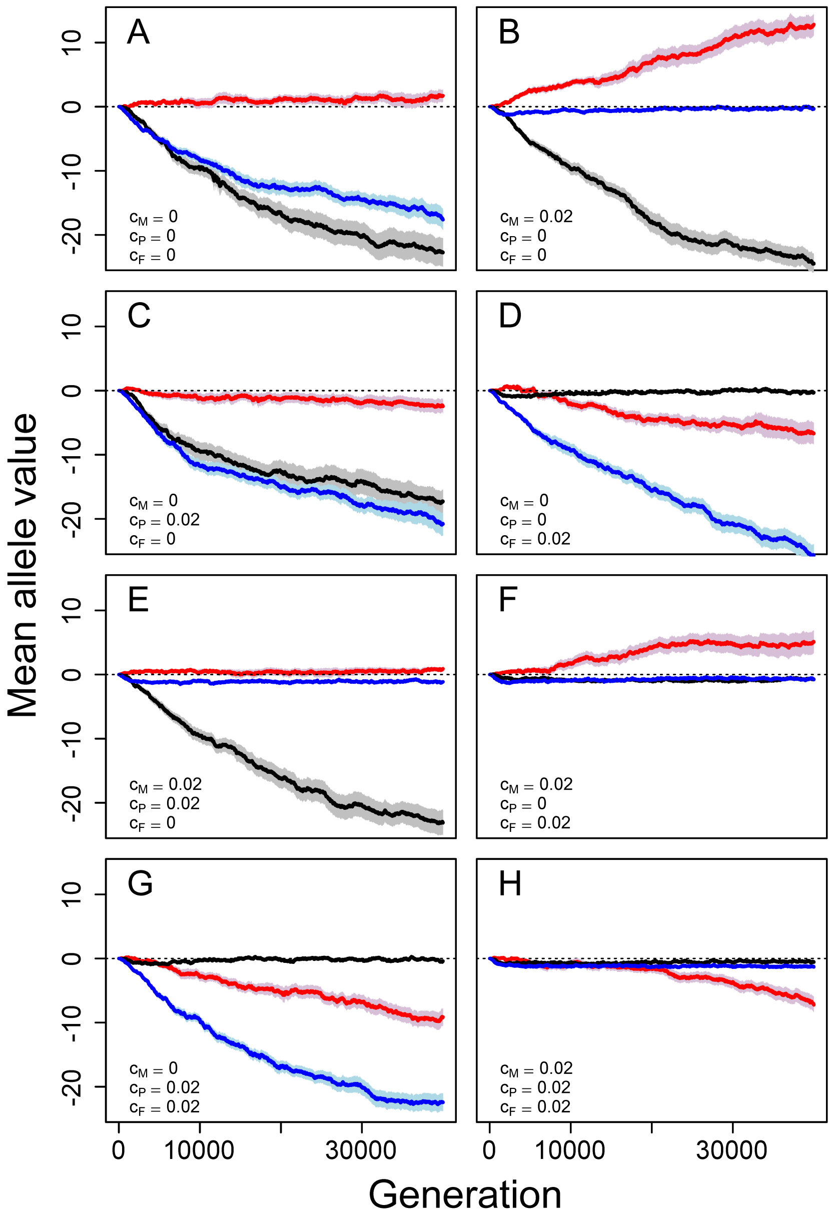

Figure S11: Mean allele values underlying tendency for polyandry (red lines and shading), pre-copulatory inbreeding strategy (blue lines and shading), and post-copulatory inbreeding strategy (black lines and shading), as calculated across all individuals within a population over 40000 generations given strong inbreeding depression. Solid lines represent grand means over 40 replicate populations, and shading around solid lines show standard deviations of allele values around grand means. Negative mean allele values indicate inbreeding avoidance or tendency for monandry, and positive values indicate inbreeding preference or tendency for polyandry. Panels A-H show simulations for different combinations of costs for tendency for polyandry (c_P), pre-copulatory inbreeding strategy (c_M), and post-copulatory inbreeding strategy (c_F). Dotted horizontal black lines in panels show values of zero on the y-axis. Individual replicates for all cost combinations are shown in Figures S1-S8.

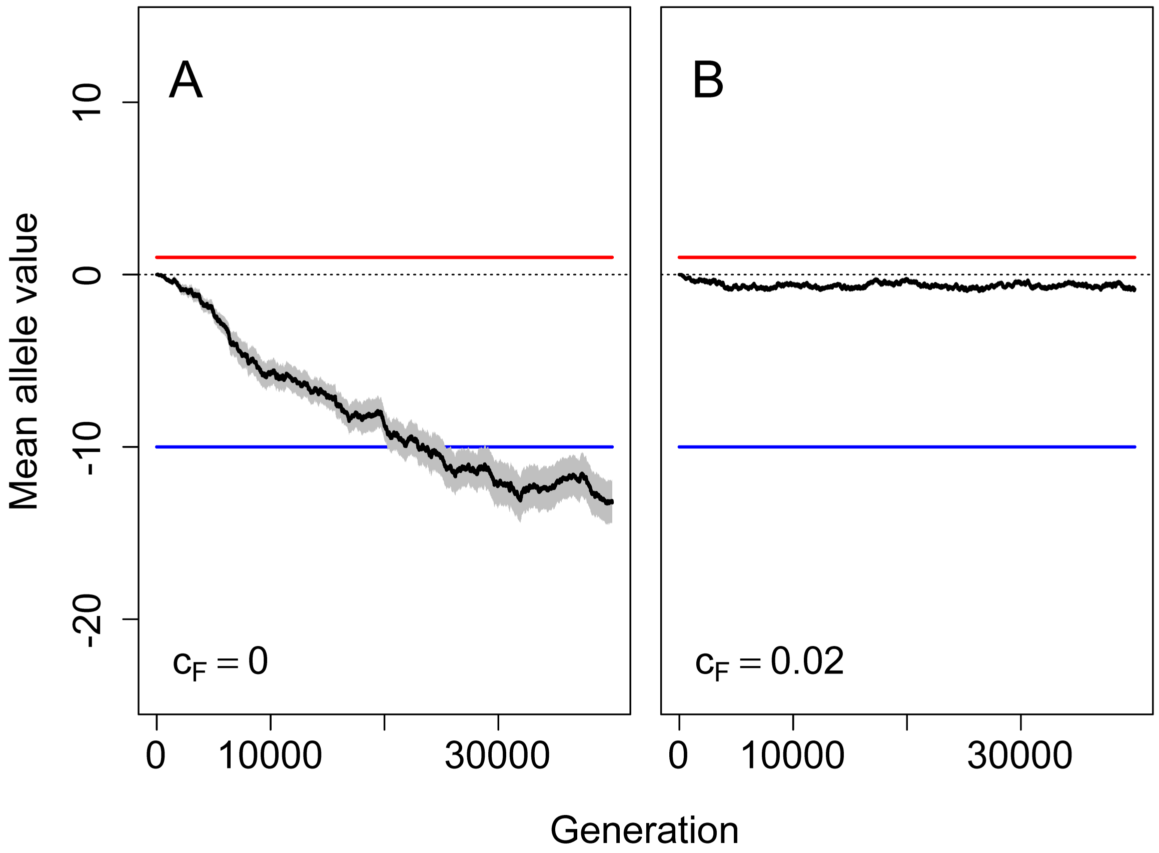

Figure S12: Mean allele values underlying post-copulatory inbreeding strategy (i.e., black lines and shading), as calculated across all individuals within a population over 40000 generations given strong inbreeding depression. Solid black lines represent grand means over 40 replicate populations, and grey shading around solid lines show standard errors of allele values around grand means. Negative mean allele values indicate strategies of inbreeding avoidance, and positive values indicate strategies of inbreeding preference. Red and blue horizontal lines indicate fixed polyandry allele values of $P_a = 1$ and fixed pre-copulatory inbreeding strategy allele values of $M_a = -10$, respectively. Panels (A) and (B) show simulations given not costly and costly post-copulatory inbreeding strategy, respectively. Dotted horizontal lines show where the y-axis equals zero.
